# Soft tissue atrophy following intralesional corticosteroid injection for infantile hemangioma: A retrospective cohort study

**DOI:** 10.1016/j.jdin.2025.11.003

**Published:** 2025-11-25

**Authors:** Jie Jiang, Rui Chang, Jingjing Sun, Shih-Jen Chang, Lei Chang, Xiaoxi Lin, Yajing Qiu

**Affiliations:** Department of Plastic and Reconstructive Surgery, Shanghai Ninth People's Hospital, Shanghai Jiao Tong University School of Medicine, Shanghai, China

**Keywords:** adverse effect, epidemiology, infantile hemangioma, intralesional corticosteroid injection, propranolol, recovery, soft tissue atrophy

*To the Editor:* Intralesional corticosteroid injection is a commonly used and relatively effective treatment for localized infantile hemangiomas (IHs) during the proliferative phase.^1^^,^^2^ However, their use is associated with multiple potential adverse effects, including adrenal suppression, failure to thrive, atrophy of soft tissues, and localized hypopigmentation or depigmentation.^3^^,^^4^ While some effects may resolve spontaneously, the long-term course and completeness of soft tissue recovery remain unclear. This study aims to evaluate recovery outcomes of soft tissue atrophy in children with IH following intralesional corticosteroid therapy.

We conducted a retrospective chart review at the Vascular Anomalies Center, Shanghai Ninth People’s Hospital, with institutional review board approval. Ten infants with localized IH who developed soft tissue atrophy after intralesional compound betamethasone injections between July 2023 and March 2025 were included. Dose was lesion-based (≤1 mL per session) with ≥1-month intervals. Demographic and clinical variables—including age, sex, lesion site, onset and resolution of atrophy, and associated complications—were extracted from electronic medical records. Descriptive statistics were applied to summarize patient characteristics and clinical outcomes.

Ten infants (2 males, 8 females) were analyzed, with a median age at first injection of 5 months (range, 1.5-15 months). Each patient received 1-3 sessions, with per-session volumes ranging from 0.1 to 0.9 mL. All lesions were focal-type, most frequently involving the scalp, abdominal wall, chest, shoulder, and upper lip.

Soft tissue atrophy developed in all patients, presenting as localized depressions at injection sites within 2 weeks to 3 months after the last injection. Seventy percent occurred within the first month (Fig 1). Recovery, defined as restoration of normal contour, was observed in all cases, with durations ranging from 1 to 12 months (median, 7 months) (Fig 2). Recovery time showed no consistent correlation with injection number, per-session dose, or lesion site. For example, a patient with a single 0.1 mL injection required 12 months for resolution, whereas another who received 3 injections (totaling 2.2 mL) recovered within 3 months. Other adverse events were infrequent and mild, including transient postinjection irritability (*n* = 1) and growth retardation (*n* = 1).

**Fig 1:**
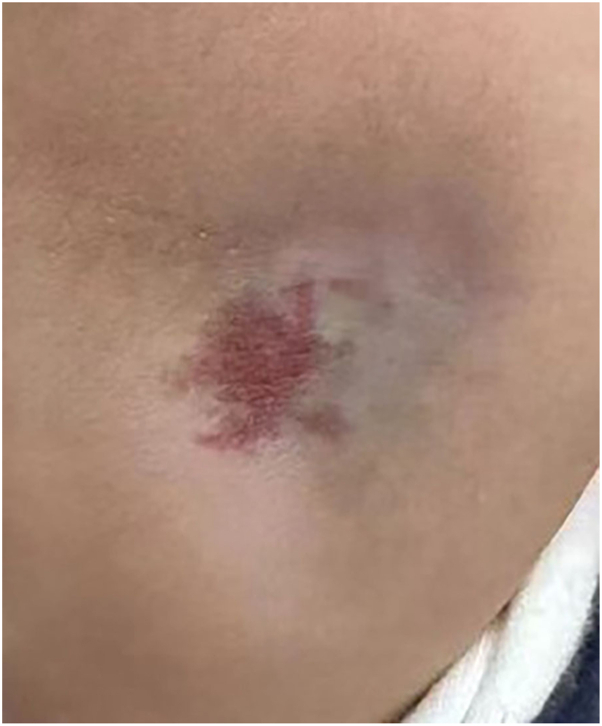
Clinical photograph 4 months after intralesional compound betamethasone injection, showing pronounced soft tissue atrophy at the injection site.

**Fig 2:**
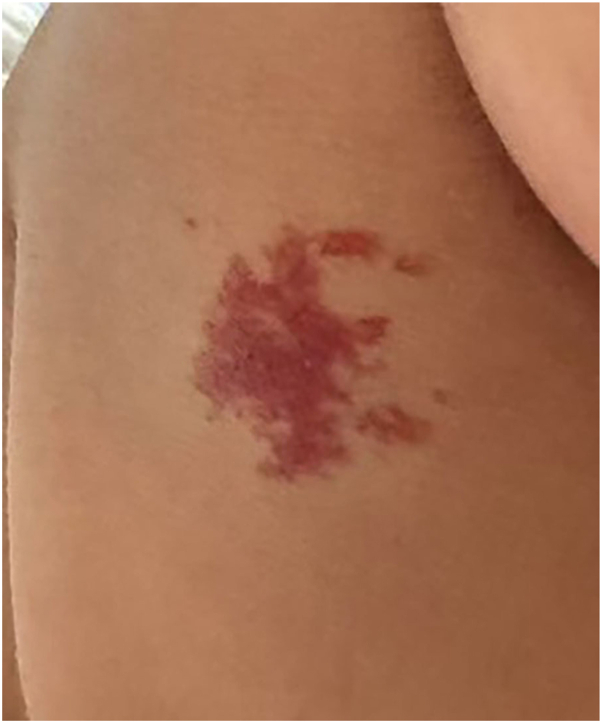
Clinical photograph 10 months after injection, showing significant improvement with restoration of the skin contour to near normal.

Although the precise mechanism was not assessed in this study, soft tissue atrophy likely results from reduced collagen synthesis, impairing normal skin structure and contributing to localized depressions.^5^ Limitations include the retrospective design and small sample size, as well as reliance on clinical photographs rather than precise volumetric imaging. Ultrasound-based volumetric data weren’t available, precluding assessment of the dose–volume relationship. Histological confirmation was not performed. Finally, findings are derived from a single-center cohort, which may limit generalizability.

In conclusion, intralesional corticosteroid injection is a safe and effective option for small, localized IHs, especially when β-blockers are contraindicated or ineffective. Soft tissue atrophy is the most common adverse effect, but it is typically temporary and resolves spontaneously. Corticosteroid-induced atrophy may form a depression below the surrounding skin, whereas natural involution of hemangiomas does not, helping clinicians distinguish the 2 during follow-up. Clinicians can be reassured that localized atrophy at the injection site generally recover without intervention, and should avoid unnecessary procedures.

## Conflicts of interest

None disclosed.

## References

[1] Couto J.A., Greene A.K. (2014). Management of problematic infantile hemangioma using intralesional triamcinolone: efficacy and safety in 100 infants. J Plast Reconstr Aesthet Surg.

[2] Yuan S.M., Zhang M., Guo Y., Cui L., Hong Z.J., Jiang H.Q. (2015). Intralesional injection of diprospan is effective for infantile hemangioma. J Craniofac Surg.

[3] Goyal R., Watts P., Lane C.M., Beck L., Gregory J.W. (2004). Adrenal suppression and failure to thrive after steroid injections for periocular hemangioma. Ophthalmology.

[4] Emir S., Gürlek Gökçebay D., Demirel F., Tunç B. (2015). Efficacy and safety of intralesional corticosteroid application for hemangiomas. Turk J Med Sci.

[5] Oikarinen A., Autio P. (1991). New aspects of the mechanism of corticosteroid-induced dermal atrophy. Clin Exp Dermatol.

